# 6′-O-galloylpaeoniflorin regulates proliferation and metastasis of non-small cell lung cancer through AMPK/miR-299-5p/ATF2 axis

**DOI:** 10.1186/s12931-020-1277-6

**Published:** 2020-02-03

**Authors:** Jinying Gao, Lei Song, Huan Xia, Liping Peng, Zhongmei Wen

**Affiliations:** Department of Respiratory Medicine, Key Laboratory of Organ Regeneration and Transplantation of the Ministry of Education, First Hospital of Jilin University, Changchun, Jilin Province China

**Keywords:** microRNA, Non-small cell lung cancer, ATF2; metastasis, Proliferation

## Abstract

**Background:**

Recent studies have shown 6'-O-galloylpaeoniflorin (GPF), a nature product extracted from the roots of paeoniflorin exerts anti-oxidant and anti-inflammatory activities. However, the effects of GPF on the proliferation and invasion in non-small cell lung cancer (NSCLC) cells have not been clarified.

**Methods:**

MTT assay was performed to determine the cytotoxicity of GPF treatment on NSCLC cells. Colony formation assay, cell scratch test and transwell assay were performed to determine the proliferation and invasion of NSCLC cells in vitro, respectively. An A549 cell xenograft mouse model was performed to confirm the growth of NSCLC cells in vivo. Western blotting was used to measure the levels of activating transcription factor 2 (ATF2), AMP-activated protein kinase (AMPK) and phosph-AMPK (p-AMPK). Luciferase assay was used to validate the binding of miR-299-5p on the 3' untranslated region (UTR) of ATF2.

**Results:**

Administration of GPF (50 or 100 μM) was significantly cytotoxic to A549 cells and H1299 cells, as well as inhibited the clonality, invasion and metastasis of NSCLC cells in vitro. GPF treatment also inhibited the tumor growth of NSCLC cell mouse xenografts in vivo. Exotic expression of miR-299-5p significantly inhibited the growth of NSCLC cells in vitro *and* in vivo. Downregulation of miR-299-5p expression attenuated the inhibition of the proliferation and metastasis of non-small cell lung cancer cells by GPF treatment. miR-299-5p significantly decreased ATF2 mRNA and protein levels in A549 cells (*p* < 0.05). Overexpression of ATF2 blocked the inhibitory effect of miR-299-5p on the proliferation and invasiveness of A549 cells.

**Conclusions:**

GPF regulates miR-299-5p/ATF2 axis in A549 cells via the AMPK signalling pathway, thereby inhibiting the proliferation and metastasis of non-small cell lung cancer cells.

## Background

Lung cancer causes widespread morbidity, and more advanced treatments are desperately needed. In a global survey of lung cancer, the incidence of non-small cell lung cancer (NSCLC) was found to be ~ 85% [1], which is significantly higher than small cell lung cancer. First-line and traditional treatments for non-small cell lung cancer include chemotherapy, radiotherapy and surgery. In the past decade, targeted therapy and tumour immunotherapy have arisen as new treatments for non-small cell lung cancer, and these treatments have rapidly progressed [1]. However, in clinical treatment, the availability of new drugs has increased treatment costs, and adverse side effects have been reported in some cases [2]. Therefore, it is still necessary to develop new drugs for the treatment of cancer patients, and to study the exact mechanisms of drugs.

MicroRNAs (miRNAs) are a class of small non-coding RNAs of 18–25 nt that inhibit target gene expression by directly binding to the 3′-UTR of RNAs [3, 4]. It is estimated that more than 60% of human protein-coding genes are regulated by miRNAs [5]. MiRNAs play a role in many physiological and pathophysiological processes, such as embryonic development [5], stem cell differentiation [6], inflammation [7] and cancer [8]. Additionally, accumulating evidence suggests that miRNAs perform key regulatory roles in various types of malignancies, including non-small cell lung cancer. For example, miR-205 targets the tumour suppressor factor SMAD4 to accelerate cell cycle progression in non-small cell lung cancer cells [9]. In addition, our previous studies showed that both the decreased expression of miR-126 in A549 cells and the upregulation of miR-126 inhibited the proliferation, migration and invasion of A549 cells by targeting phosphatidylinositol 3-kinase regulatory subunit beta (PIK3R2) [10]. miRNAs also play an important role in the sensitivity of non-small cell lung cancer cells to chemotherapy and radiotherapy by regulating DNA repair genes [11, 12]. Therefore, miRNAs are potential biomarkers for the diagnosis, prognosis, prevention and treatment of non-small cell lung cancer. Many natural Chinese herbal ingredients have been found to regulate microRNAs, such as *Lycium barbarum* polysaccharides [13], Coptisine from Rhizoma Coptidis [14], resveratrol, curcumin and berberine [15].

6′-O-galloylpaeoniflorin (GPF) is extracted from the roots of paeoniflorin and consists of D-glucose, galloyl and benzoyl moieties [16]. D-glucose [17] exists as an open chain or ring structure with α - and β - isomers. It exists widely in the fruit of plant or animals’ body fluid in a free state, and as a component of polysaccharide and glucoside in nature. It can be used as reductant in industry. And for benzoyl moieties and galloyl groups, they present in several natural tea catechins, are responsible for most of their antioxidant, anticancer and antimicrobial activities [18–20]. Studies have shown that GPF has significant antioxidant activity [21, 22], but its role in the growth and metastasis of tumour cells is not fully understood. The present study focused on the effects of GPF on the biological functions of non-small cell lung cancer and its potential molecular mechanisms, with the aim of providing more options for the clinical treatment of lung cancer.

## Materials and methods

### Cell culture and treatment

Normal human airway epithelial Beas 2B and 16-HBE cells and NSCLC cell lines (A549 and H1299 cells) were purchased from the American Type Culture Collection (Manassas, VA, USA). The above cells were cultured with Dulbecco’s modified Eagle medium (DMEM; Gibco) supplemented with 10% fetal bovine serum (FBS, Gibco) and 4 mM glutamine at 37 °C under 5% CO2 conditions.

For inhibition of AMP-activated protein kinase (AMPK) pathway, A549 and H1299 cells were pre-treated with Compound C (a specific inhibitor of AMPK), and divided into control (not treated), agomiR-299-5p group (transfected with agomiR-299-5p) and agomir-NC group (transfected with agomir-NC) groups.

A549 cells were divided into control (without treatment), mimics + vector (A549 cells transfected with agomiR-299-5p and empty plasmid) and mimics + ATF2 (A549 cells transfected with agomiR-299-5p and ATF2 overexpression plasmid) groups.

### MTT assay

A549, H1299, Beas 2B and 16-HBE cells at a density of 2 × 10 ^4^ cells/ml were respectively seeded in 96-well plates with 200 μl in each well. After treatment, cells were incubated in an incubator (37 °C, 5% CO_2_) with 20 μL of 3-(4,5-dimethylthiazol-2-yl)-2,5-diphenyltetrazolium bromide solution (MTT) (5 mg/mL) for 4 h. The medium containing MTT solution was removed, and 200 μL of dimethyl sulfoxide was added. The spectrophotometric absorbance at 490 nm was determined using a microplate reader (Bio-Rad, PA, USA). Each experiment was performed in triplicate. Cell survival rate was then calculated using the equation: Cell survival rate (%) = (Values for the experimental group/Values for the control group) × 100%.

### Colony formation assay

Cells were plated on 3.5-cm plates and cultured overnight followed by the addition of DMEM medium. The medium was changed once every 72-h followed by the addition of GPF. After cell culture for 2 weeks, the supernatant was removed, and 20% formaldehyde was added. After 15 min, 0.1% Crystal Violet staining was performed. Three visual fields at 10 times of magnification were selected under an optical microscope and cells were counted in triplicate.

### Tumour-bearing mouse model

Specific Pathogen Free (SPF)-grade healthy BALB/C-nu/nu nude mice aged 5 to 7 weeks (15 male and 15 female) weighing 15–25 g was randomly divided into control, 5 mg/kg GPF, and 10 mg/kg GPF treatment groups. Exponentially growing cells (1 × 10^6^ cells/ml, 100 μl) in 0.2 mL of phosphate-buffered saline (PBS) was injected subcutaneously into both sides of the posterior buttocks of nude mice. Seven days after cell injection, PBS (20 μL), GPF (5 or 10 mg/kg) was intratumorally injected into the implanted tumour. Tumour volumes were measured with vernier calipers every 7 days. In the second part, 7 days after cell injection, PBS (20 μL), agomir-299-5p (1 nmol in 20 μL of PBS for each mouse), or an equivalent amount of agomir-NC (RiboBio) was intratumorally injected into the implanted tumour every 3 days. Tumour volumes were measured with vernier calipers every 3 days, and the tumour volume was calculated as follows: tumour volume (mm^3^) = maximal length (mm) × [perpendicular width (mm)]^2^ / 2. The tumours were removed and photographed on day 28. Each group had at least six mice.

### Cell scratch test

Cells were cultured in 6-well plates. When the cells grew to 90% confluency, the surface of the plate was scratched along a marked line; the cells were rinsed with PBS three times to wash off the floating cells. The cells were then cultured in DMEM without FBS at 37 °C in a 5% CO2 incubator with saturated humidity. The cells were photographed under an inverted microscope at 0, 6, 12 and 24 h, respectively. Imagetool software (Bechtel Nevada, Los Alamos Operations) was used to calculate the healing area, and the healing rate was calculated as follows: (initial scratch width – existing scratch width)/initial scratch width × 100%.

### Transwell assay

A 200 μl sample of cells (1–10 × 10^5^) were added to the upper chamber in serum-free DMEM, and 500 μl of DMEM containing 10% FBS was added to the lower chamber of a 24-well plate. After incubation for 24 h at 37 °C with 5% CO2, the number of cells that had migrated through the pores was quantified by counting five random fields under a microscope (Olympus, Tokyo, Japan). The same experiments were independently repeated three times. The procedure for the transwell invasion assay was the same as that of the transwell assay, except that the filter in the top chamber was coated with Matrigel.

### Detection of AMPK, p-AMPK and ATF2 expression by western blotting

The total protein from cells was extracted using RIPA buffer (Pierce, Rockford, IL, USA). The protein concentration was determined using the Bradford method (Pierce). Equal amounts of proteins (40 μg) were separated on 10% sodium dodecyl sulfate-polyacrylamide gels and transferred to Immobilon-P membranes (Millipore, Bedford, MA, USA). After blocking with 5% nonfat milk in phosphate-buffered saline (PBS)–Tween-20 for 1 h, the membranes were incubated with anti-RAB14, anti-Akt, anti-p-Akt, anti-CCND1, anti-CDK2, or anti-Bax-antibody (1:2000 dilution, Abcam, Hong Kong, China), as well as anti-GAPDH (1:2000, Abcam) antibody at 4 °C overnight, followed by incubation with horseradish peroxidase-conjugated secondary antibody (1:5000) at 37 °C for 1 h. Immunoreactive bands were detected using the ECL Plus Detection kit (Pierce, Rockford, IL, USA).

### MicroRNA transfection into A549 cells

The miR-299-5p mimic and scrambled control miRNA were purchased from RiboBio Co. (Guangzhou, China) and transfected into cells using riboFECT™ CP Reagent (RiboBio), according to the manufacturer’s protocol. ATF2 overexpression particles were obtained from Hanheng (Shanghai, China) and transduced with 5 mg/mL polybrene (Sigma-Aldrich, St. Louis, MO, USA). Stable cell lines were selected with puromycin (Santa Cruz Biotechnology, Santa Cruz, CA, USA).

### Luciferase assay

The online database TargetScan (http://www.targetscan.org/) was used to search for potential miR-299-5p targets. ATF2 was found to be one of the predicted miR-299-5p targets. In order to verify ATF2 as an authentic miR-299-5p target, the pMIR-REPORT vector was reconstructed by inserting a luciferase reporter vector with the DNA sequence of the ATF2 3′UTR containing the putative miR-299-5p-binding site, generating pMIR-REPORT-ATF2–3′UTR-wt. A luciferase vector harboring a mutant miR-299-5p-binding site was also constructed and used as a control (pMIR-REPORT-ATF2–3’UTR-mut). The luciferase reporter vectors and miR-299-5p mimic were transfected into HEK-293 T cells using LipofectamineTM 2000 (Invitrogen), followed by the detection of luciferase activity with a Dual-Luciferase® Reporter Assay kit (Promega, USA).

### Detection of ATF2 mRNA by quantitative real-time PCR (qRT-PCR)

Total RNA was extracted from tissues using TRIzol reagent (Invitrogen, Carlsbad, CA, USA) and used as a template for cDNA synthesis using the EasyScript First-Strand cDNA Synthesis SuperMix (TransGen Biotech, Beijing, China). qRT-PCR was performed using the TransStart™ SYBR Green qPCR Supermix (TransGen Biotech) on a 7300 PCR System (ABI, Carlsbad, USA). The primers for ATF2 mRNA was purchased from RiboBio. The relative expression levels of mRNA were calculated through 2^-ΔΔCt^ method. The primer sequences were as the follows: ATF2: F: 5′- CCGGATCCATGAAATTCAAGTTACATGT-3′, R: 5′-GGCTCGAGTCAACTTCCTGAGGGCTGTG-3′.

### Plasmid transfection

Carrier and packaging plasmids containing ATF2 genes were co-transfected into HEK293 cells which were infected with adenovirus by the calcium phosphate co-transfection method. The crude extract of recombinant adeno-associated virus (rAAV) was purified by caesium chloride equilibrium gradient centrifugation, and the viral titre was determined.

### Statistical analysis

Statistical Program for Social Sciences 21.0 software (SPSS, Inc., Chicago, IL, USA) was used for statistical analysis of the collected data. Measurement data are expressed as mean ± standard deviation (SD), and were compared by t-test (between two groups) and variance analysis (multiple groups). A *p*-value < 0.05 was considered to indicate statistically significant differences, and *p* < 0.01 was considered to indicate highly statistically significant differences.

## Results

### GPF was cytotoxicity activity to non-small cell lung cancer cells in vitro

**Fig. 1:**
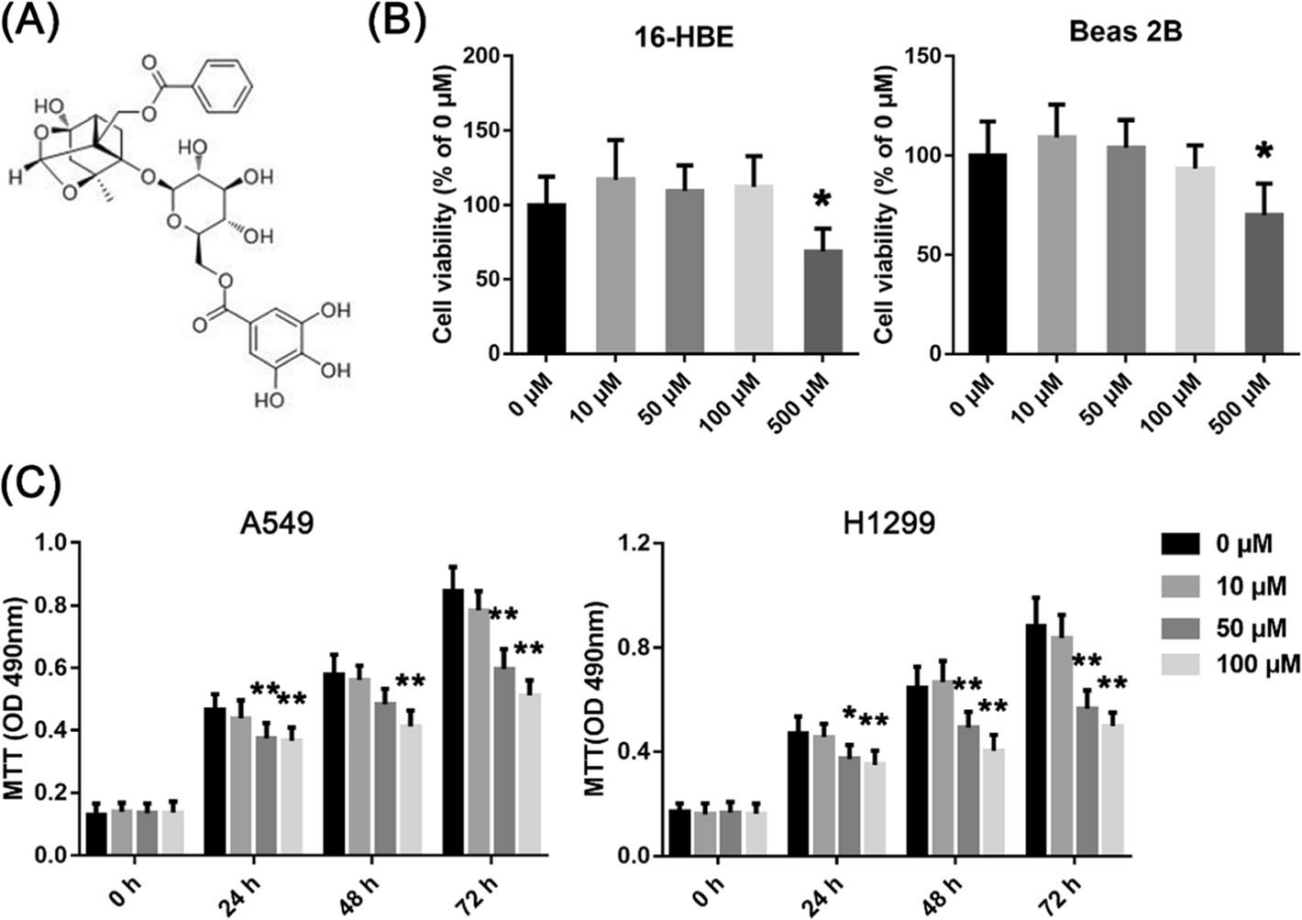
A549, H1299, 16-HBE and Beas 2B cells were treated with different concentrations of GPF, and MTT assays were performed. **a** The chemical structural formula of GPF. **b** GPF did not inhibit the activity of Beas 2B or 16-HBE human normal epithelial cell lines at 50 or 100 μM. **c** GPF had significantly cytotoxicity activity on A549 or H1299 human non-small cell lung cancer cell lines in vitro at GPF concentrations of 50 or 100 μM. The volume of tumours in the control group was significantly larger than that in treatment groups from day 21

**Fig. 2:**
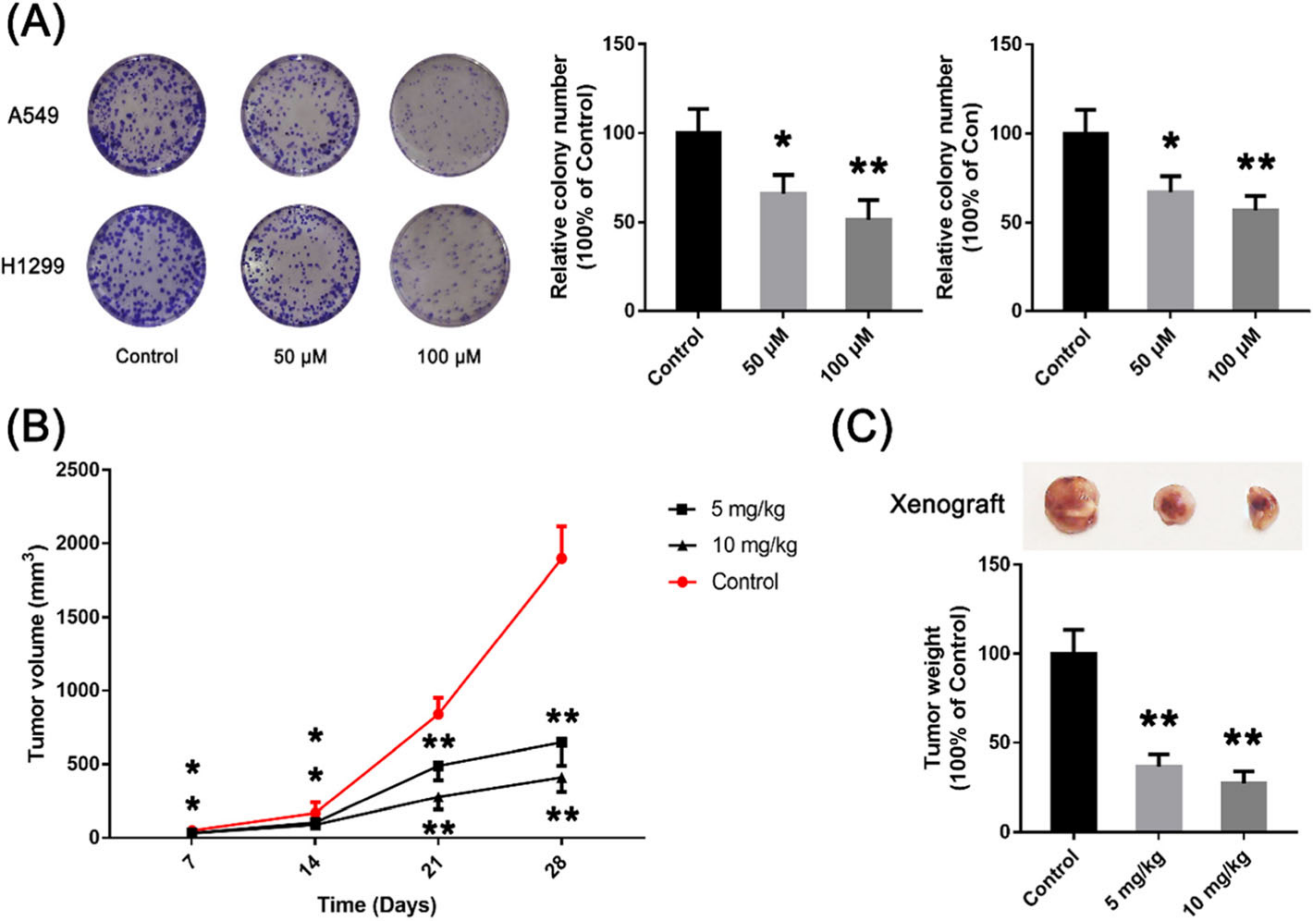
Effects of GPF on the formation and growth of non-small cell lung cancer cells in vivo*.* **a** GPF (50 or 100 μM) inhibited the cloning ability of A549 and H1299 cells in vitro. **b** The volume of tumours in the control group was significantly larger than in treatment groups from day 21. **c** The weight of tumours was also higher in the control group than in treatment groups from day 21

In this experiment, the absorbance of A549 and H1299 cells decreased significantly at 490 nm at both GPF concentrations (50 and 100 μM) at 24–72 h (Fig. 1c; Table 1). However, the absorbance of Beas 2B and 16-HBE cells remained unchanged at 490 nm at both GPF concentrations (50 and 100 μM) at 24–72 h, and the absorbance of normal cells decreased when the GPF concentration was 500 μM (Fig. 1b). The results of clonal formation assays showed that GPF treatment significantly reduced the number of colonies in a dose-dependent manner when compared with the untreated cells in vitro (Fig. 2a; Table 2). These results indicated that GPF has cytotoxicity activity to A549 and H1299 cells.

**Table 1:** The absorbance of A549 and H1299 cells at 490 nm at different GPF concentrations (50 and 100 μM) at 24–72 h

Absorbance/GPF concentrations/Test time	The absorbance of A549 cells				
	0 μM	50 μM	100 μM	*P* value (0 μM vs 50 μM)	*P* value (0 μM vs 100 μM)
0 h	0.131	0.136	0.139	0.7926	0.6897
24 h	0.466	0.374	0.366	0.0097	0.0043
48 h	0.578	0.483	0.412	0.0191	0.0007
72 h	0.846	0.597	0.512	0.0001	0.0000
Absorbance/GPF concentrations/Test time	The absorbance of H1299 cells				
	0 μM	50 μM	100 μM	*P* value (0 μM vs 50 μM)	*P* value (0 μM vs 100 μM)
0 h	0.169	0.166	0.162	0.8888	0.7331
24 h	0.470	0.372	0.351	0.0161	0.0059
48 h	0.645	0.491	0.403	0.0042	0.0002
72 h	0.881	0.565	0.497	0.0002	0.0000

**Table 2:** The relative colony number of A549 and H1299 cells at different GPF concentrations (50 and 100 μM)

	The relative colony number of A549 cells				
	0 μM	50 μM	100 μM	*P* value (0 μM vs 50 μM)	*P* value (100 μM vs 50 μM)
mean	100	66.2	51.3	0.0256	0.1666
	The relative colony number of H1299 cells				
	0 μM	50 μM	100 μM	*P* value (0 μM vs 50 μM)	*P* value (100 μM vs 50 μM)
mean	100	67.2	56.8	0.0230	0.2108

The results of tumour-bearing mouse model showed that the volume of tumours in the control group was significantly larger than treatment groups from day 21 (Fig. 2b; Table 3), and the weight of tumours was also higher in the control group than in treatment groups (Fig. 2c; Table 3). These results suggest that GPF can inhibit tumorigenesis and the growth of non-small cell lung cancer cells in vivo.

**Table 3:** The tumor volume and tumor weight in different groups at 7–28 days

	Tumor volume (mm^3^)					
	control	5 mg/kg	10 mg/kg	*P* value (control vs 5 mg/kg)	*P* value (control vs 10 mg/kg)	*P* value (5 mg/kg vs 10 mg/kg)
Day 7	49.3	32.2	30.7	0.0221	0.0164	0.7382
Day 14	167.9	104.5	87.9	0.0662	0.0245	0.1636
Day 21	839.3	488.7	277.2	0.0002	0.0000	0.0024
Day 28	1899	647.9	409.3	0.0000	0.0000	0.1084
	Tumor weight (g)					
Day 28	100	36.8	27.2	0.0000		0.0346

### GPF inhibits invasion and metastasis of non-small cell lung cancer cells in vitro

**Fig. 3:**
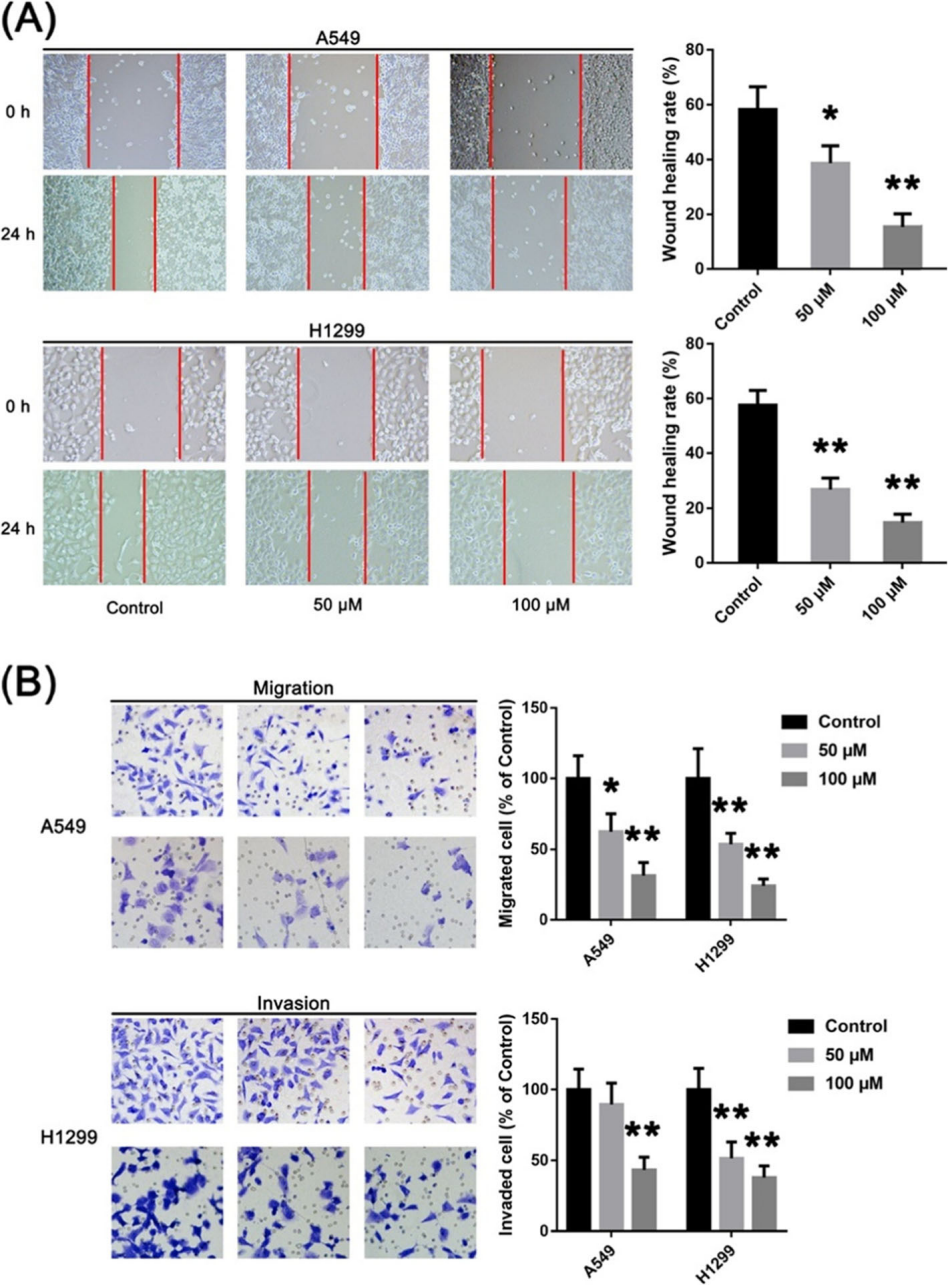
Effects of GPF on migration and invasion of non-small cell lung cancer cells in vitro. **a** The results of scratch tests of non-small cell lung cancer cells treated or untreated with GPF. **b** The results of Transwell assays of non-small cell lung cancer cells treated or untreated with GPF

The results of scratch tests showed that the migration distance of A549 cells treated with GPF was significantly less than that of control cells after 48 h of culture (Fig. 3a). Furthermore, the number of A549 cells passing through the gel matrix and filter membranes following GPF administration was significantly less than that in the control group in Transwell assays. These results suggest that GPF can inhibit the migration and invasion of non-small cell lung cancer cells (Fig. 3b).

### GPF up-regulates miR-299-5p expression via the AMPK pathway

**Fig. 4:**
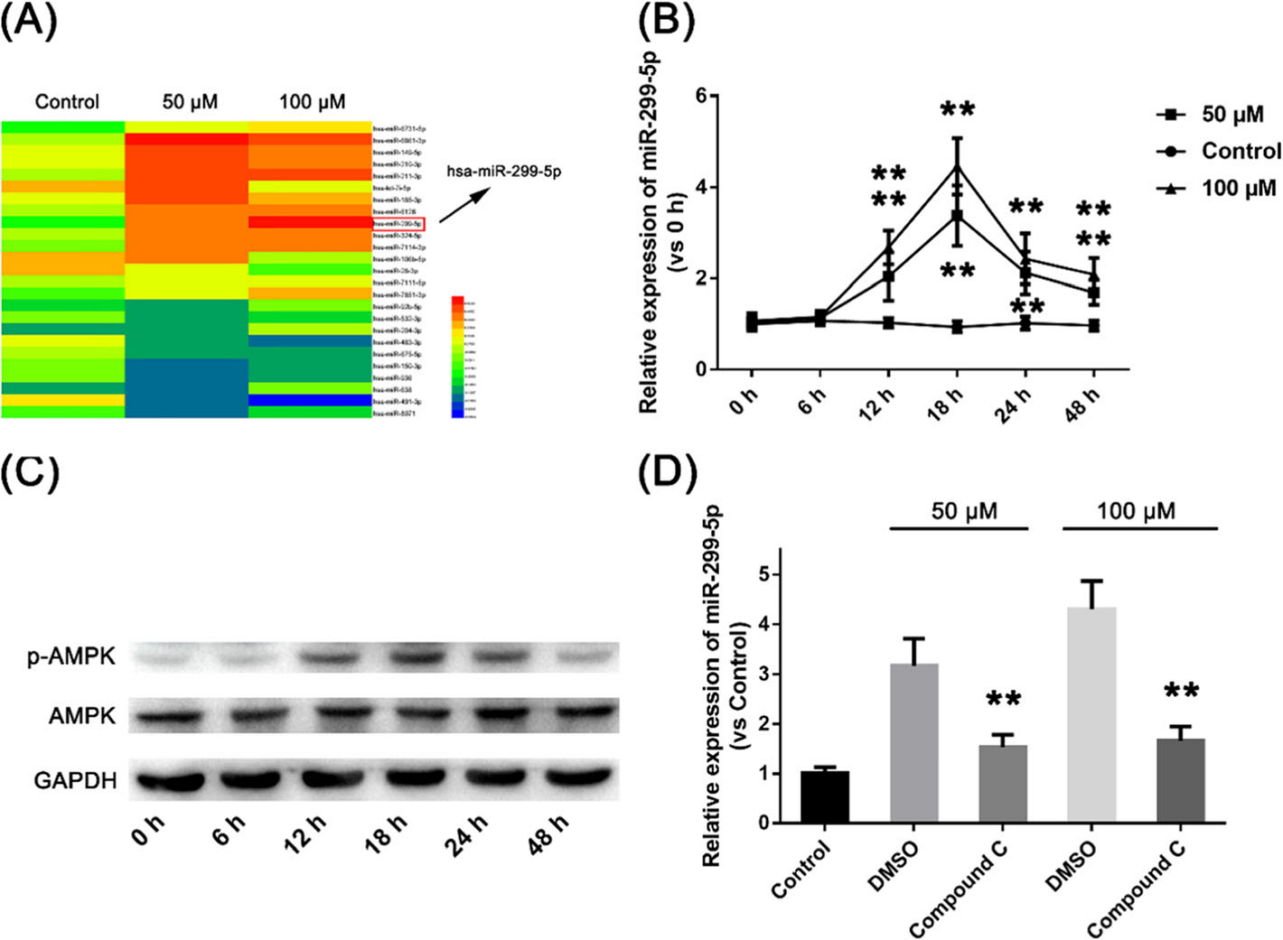
**a** Comparison of differentially expressed microRNAs in stimulated and unstimulated cells by deep sequencing. **b** Expression of miR-299-5p at different concentrations of GPF (50 and 100 μM) at different timepoints assessed by qRT-PCR. **c** GPF (100 μM) stimulation significantly activates the AMPK pathway in A549 cells. **d** Levels of microRNA-299-5p in A549 cells pretreated with Compound C are significantly lower than in untreated cells after GPF stimulation

We compared differentially expressed microRNAs in stimulated and unstimulated cells by deep sequencing. The results showed that ~ 67 miRNAs in A549 cells were up-regulated by more than 2-fold, while 34 miRNAs were downregulated by 2-fold after 12 h of GPF stimulation (Fig. 4a; data not fully listed). We selected miR-299-5p for further study since this miRNA was altered significantly before and after stimulation by GPF. We tested the expression of miR-299-5p at different concentrations of GPF (50 and 100 μM) at different timepoints by qRT-PCR. The results showed that expression of microRNA-299-5p began to increase after 12 h of stimulation by GPF, and levels peaked at 18 h, then declined, but remained higher than those of cells not stimulated by GPF at 24 h (Fig. 4b; Table 4). In this study, GPF (100 μM) stimulation significantly activated the AMPK pathway in A549 cells (Fig. 4c). We pre-treated A549 cells with Compound C before GPF stimulation at different concentrations to determine whether GPF regulates the expression of microRNA-299-5p by activating AMPK. The results showed that the level of microRNA-299-5p in A549 cells pre-treated with Compound C was significantly lower than that in untreated cells after GPF stimulation (Fig. 4d). This suggests that AMPK is the signal pathway through which GPF up-regulates the expression of microRNA-299-5p.

**Table 4:** The relative expression of miR-299-5p at different GPF concentrations (50 and 100 μM) at 0–48 h

	miR-299-5p expression			
	control	50 μM	100 μM	*P* value (control vs 50 μM)
0 h	1	1.03	1.07	0.7101
6 h	1.07	1.14	1.16	0.4113
12 h	1.03	2.04	2.68	0.0010
18 h	0.94	3.38	4.46	0.0000
24 h	1.02	2.12	2.43	0.0002
48 h	0.97	1.69	2.08	0.0001

### MiR-299-5p inhibits the proliferation of non-small cell lung cancer cells

**Fig. 5:**
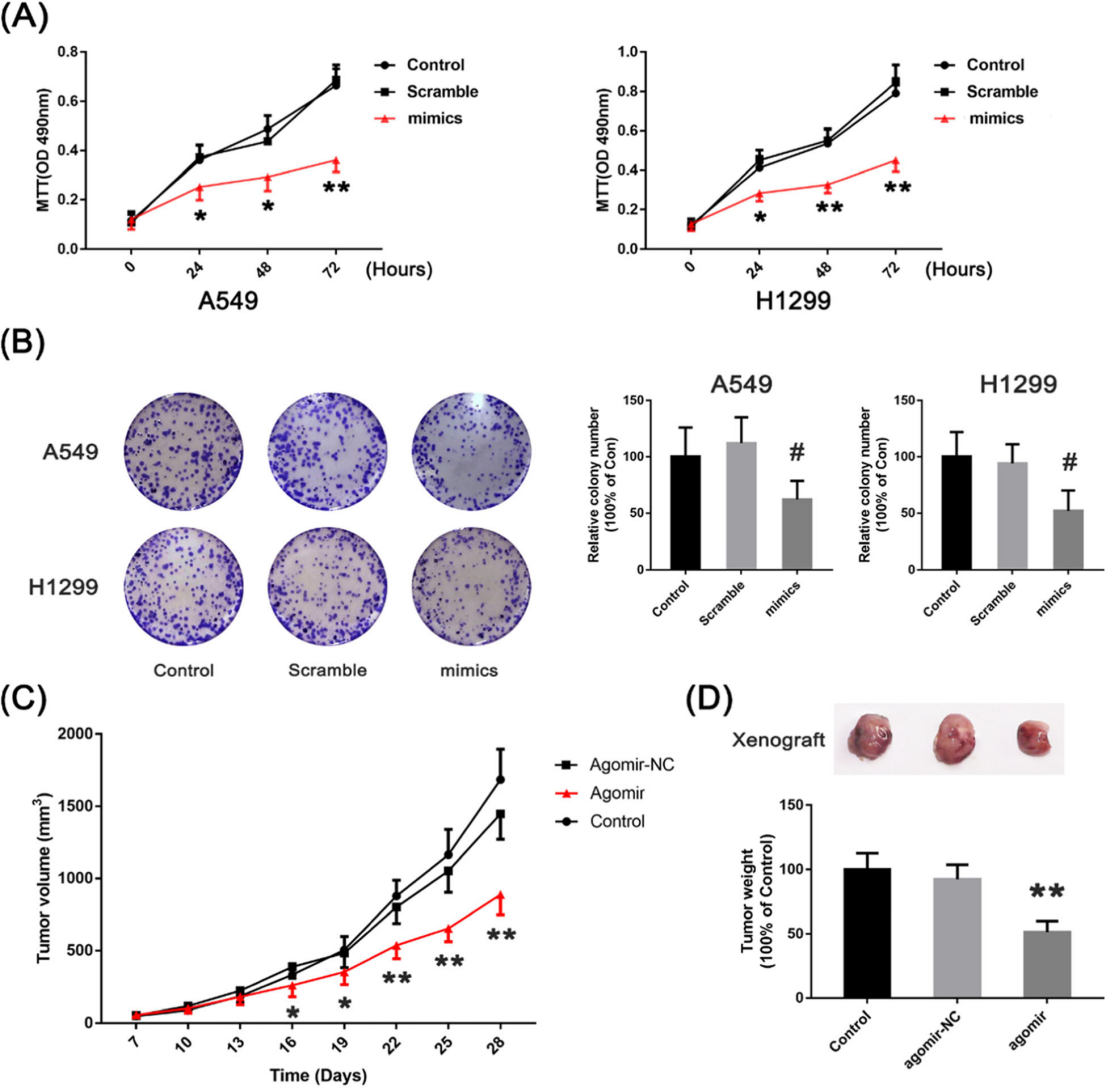
**a** Absorption changes of A549 and H1299 cells transfected with mimics at different timepoints assessed by MTT assays. **b** The number of clones of A549 and H1299 cells transfected with miR-299-5p is significantly fewer than that of the negative control group in vitro. **c**, **d** Effect of microRNA-299-5p on the growth rate of subcutaneous non-small cell lung cancer in nude mice

We transfected mimics (miR-299-5p agonist) and Scramble (its negative control) into A549 and H1299 cells and performed MTT assays every 24 h. The results showed that the absorbance at 490 nm of A549 and H1299 cells transfected with mimics was lower than that of the negative control (Scramble) group at 24 h and 48 h (*p* < 0.05), and it was significantly lower than that of the negative control group at 72 h (*p* < 0.01) (Fig. 5a; Table 5). We also carried out Clonal formation assays at the same time. The results showed that the number of clones of A549 and H1299 cells transfected with miR-299-5p was significantly less than that of the negative control in vitro (*p* < 0.01) (Fig. 5b; Table 6).

**Table 5:** The absorbance of A549 and H1299 cells at 490 nm in control, scramble and mimics (miR-299-5p agonist) group at 24–72 h

Absorbance/Groups/Test time	The absorbance of A549 cells				
	control	scramble	mimics	*P* value (control vs scramble)	*P* value (scramble vs mimics)
0 h	0.131	0.112	0.127	0.4485	0.5663
24 h	0.413	0.451	0.283	0.4457	0.1174
48 h	0.536	0.551	0.326	0.7951	0.0062
72 h	0.791	0.847	0.451	0.4464	0.0028
Absorbance/Groups/Test time	The absorbance of H1299 cells				
	control	scramble	mimics	*P* value (control vs scramble)	*P* value (scramble vs mimics)
0 h	0.122	0.112	0.127	0.6815	0.5663
24 h	0.413	0.451	0.283	0.4457	0.0117
48 h	0.536	0.551	0.326	0.7951	0.0062
72 h	0.719	0.847	0.451	0.4464	0.0028

**Table 6:** The relative colony number of A549 and H1299 cells in control, scramble and mimics (miR-299-5p agonist) group

	The relative colony number of A549 cells				
	control	scramble	mimics	*P* value (control vs scramble)	*P* value (scramble vs mimics)
mean	100	113.2	36.3	0.4620	0.0052
	The relative colony number of H1299 cells				
	control	scramble	mimics	*P* value (control vs scramble)	*P* value (scramble vs mimics)
mean	100	112.4	62.4	0.5112	0.0326

The results of the tumour-bearing mouse model showed that the volume of non-small cell lung cancer cells in the mimics group was significantly larger than that in the Scramble group from day 16 (Fig. 5c; Table 7). This suggests that miR-299-5p may inhibit the tumorigenesis and growth of non-small cell lung cancer cells in vivo.

**Table 7:** The tumor volume and tumor weight in different groups at 7–28 days

	Tumor volume (mm^3^)				
	control	AgomiR-NC	AgomiR-299-5p	*P* value (control vs AgomiR-NC)	*P* value (AgomiR-NC vs AgomiR-299-5p)
Day 7	48.6	52.1	56.6	0.7087	0.5848
Day 13	182.7	221.6	178.7	0.3628	0.3165
Day 19	504.6	483.4	351.7	0.7135	0.0374
Day 22	878.4	801.5	536.9	0.2583	0.0013
Day 28	1684	1447	887.4	0.0614	0.0001
	Tumor weight (g)				
Day 28	100	92.4	51.4	0.2971	0.0000

### Inhibitory effect of microRNA-299-5p on invasion of non-small cell lung cancer cells

**Fig. 6:**
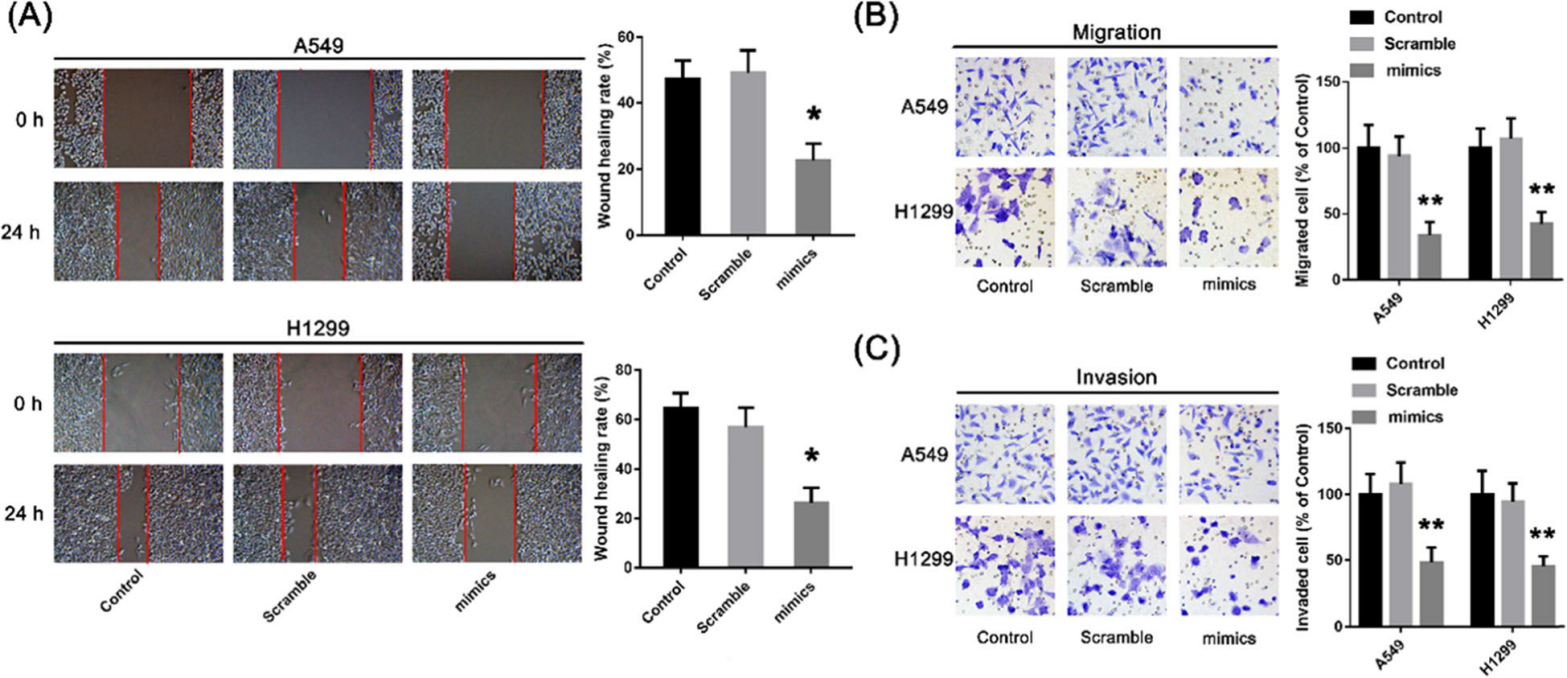
Effect of microRNA-299-5p on migration and invasion of non-small cell lung cancer cells in vitro. **a** The results of scratch test of non-small cell lung cancer cells in mimics or scramble group. **b,** **c** The results of Transwell assays of non-small cell lung cancer cells in mimics and scramble group

We used scratch test and Transwell assays to verify whether microRNA-299-5p has an effect on the invasive ability of non-small cell lung cancer cells. The results showed that the migration distance of A549 cells transfected with mimics was significantly less than that of the Scramble group after 48 h of culture (Fig. 6a). Transwell assays showed that the number of A549 cells transfected with mimics crossings through Matrigel and filter membranes was significantly fewer than that in the Scramble group. These results suggest that miR-299-5p can inhibit the migration and invasion of non-small cell lung cancer cells (Fig. 6b c).

### Downregulation of the expression of microRNA-299-5p attenuates the cytotoxicity activity of GPF to non-small cell lung cancer cells

**Fig. 7:**
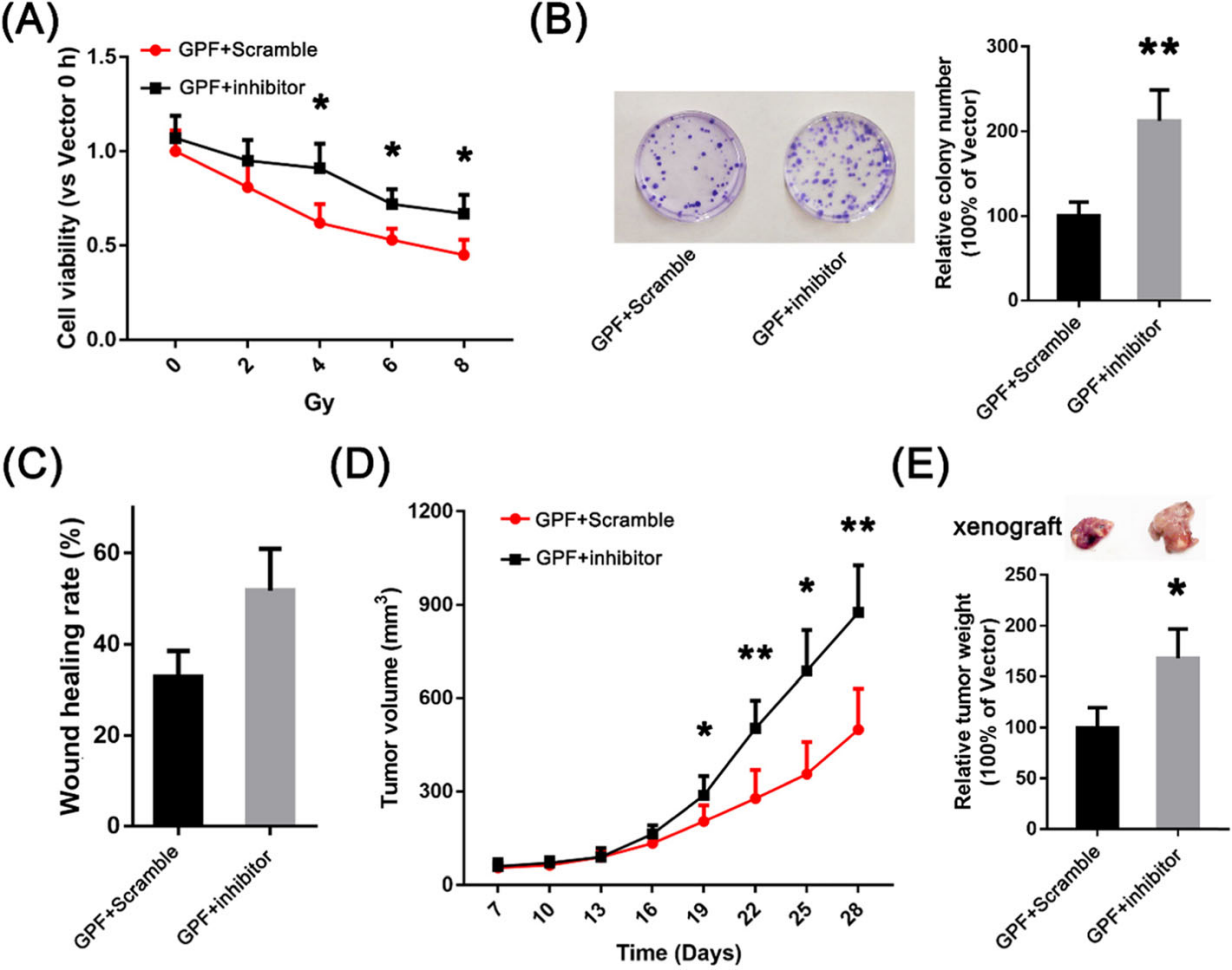
**a**-**c** The proliferation and cloning ability of A549 cells transfected with microRNA-299-5p inhibitor are significantly increased in vitro, and the ability of migration and invasion is also significantly increased in vitro. **d**, **e** The growth rate of A549 cells transfected with microRNA-299-5p inhibitor is also significantly higher than that in the GPF group in vivo

We transfected an inhibitor of microRNA-299-5p before GPF treatment of A549 cells, then measured the proliferation and metastasis of A549 cells. The results showed that the proliferation and cloning ability of A549 cells transfected with miR-299-5p inhibitor were significantly greater than those in the GPF group in vitro, and the growth rate was also significantly higher than that in the GPF group in vivo. The migration and invasion ability of A549 cells transfected with microRNA-299-5p inhibitor was also significantly higher than that of the GPF group in vitro. These results suggest that GPF acts a role of cytotoxicity and inhibits the metastasis of non-small cell lung cancer cells via the microRNA-299-5p pathway (Fig. 7).

### ATF2 is a possible target of miR-299-5p

**Fig. 8:**
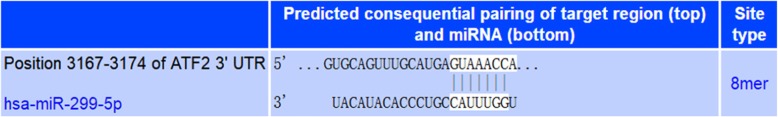
ATF2 may be a target of miR-299-5p

In total, 279 possible target genes were identified via predictions using microRNA.org, TargetScan and miRbase software. The ATF2 putative target gene attracted particular attention. The 3′-UTR region of ATF2 is 3211 nt long. At the 3′-UTR terminus (nt 3167–3174), there are 8 mer-sized fragments matching microRNA-299-5p (Fig. 8).

### MiR-299-5p down-regulates ATF2 expression in A549 cells

**Fig. 9:**
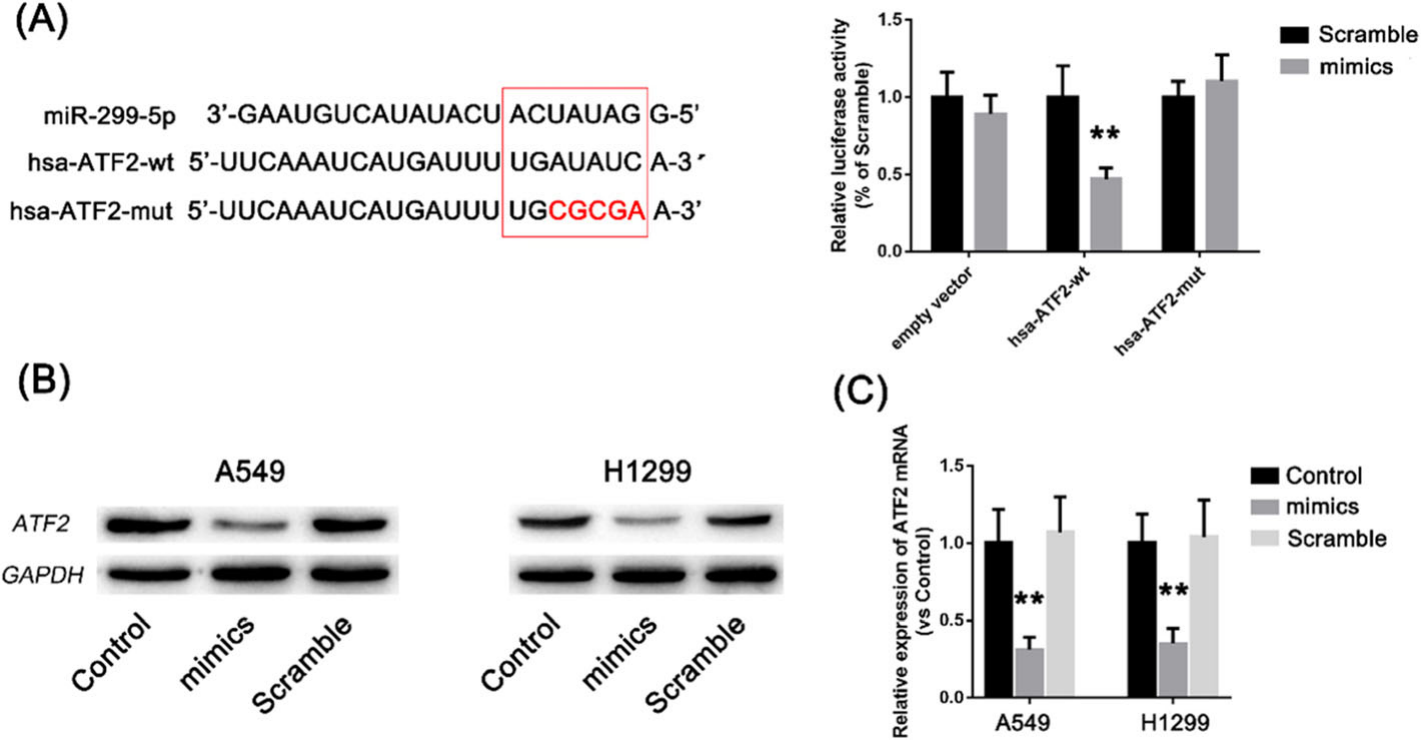
**a** ATF2–3′-UTR-wt and ATF2–3′-UTR-mut were cloned into the luciferase reporter vector. The results of luciferase assays showed that the fluorescence intensity of the ATF2–3′-UTR-wt group was significantly lower than that of the Scramble group after transfection of microRNA-299-5p mimics, but there was no significant difference between ATF2–3′-UTR-mut, empty vector or Scramble groups. **b** Western blotting showing that microRNA-299-5p mimics could significantly reduce ATF2 protein levels in A549 cells. **c** qRT-PCR showing that microRNA-299-5p mimics downregulate ATF2 expression in A549 cells. All results are expressed as mean ± SD (*p < 0.05 compared to the Scramble group, ** < 0.01 compared to the Scramble group)

In the 3′-UTR sequence of the ATF2 gene, a 7-mer base sequence matching microRNA-299-5p was identified by searching the database. The results of luciferase reporter gene assay and plasmid transfection showed that the fluorescence expression of HEK293 cells in the ATF2–3′-UTR-wt group was significantly lower than that in the Scramble group, but there was no significant difference between the fluorescence intensity of HEK293 cells in ATF2–3′-UTR-mut and blank plasmid groups (Fig. 9a). These results suggest that microRNA-299-5p may regulate the translation of ATF2 at the post-transcriptional level by binding to this predicted ATF2 mRNA 3′-UTR sequence.

To investigate the regulation of ATF2 by microRNA-299-5p at the protein level, we transfected microRNA-299-5p mimics and negative control (Scramble) into A549 cells, and measured expression of ATF2 after transfection by western blotting. The results showed that compared with Scramble, microRNA-299-5p mimics could significantly downregulate ATF2 expression in A549 cells (Fig. 9b).

We also transfected microRNA-299-5p mimics and Scramble in order to investigate whether expression of ATF2 in A549 cells was affected by microRNA-299-5p. Expression of ATF2 in A549 cells was detected by qRT-PCR. The results showed that transfection of Scramble did not alter ATF2 mRNA levels, but mimics significantly downregulated ATF2 mRNA expression (Fig. 9c). Together, these results showed that microRNA-299-5p could downregulate ATF2 protein expression by binding to the ATF2 mRNA 3′-UTR.

### Overexpression of ATF2 blocked the inhibitory effect of microRNA-299-5p on the proliferation of A549 cells

**Fig. 10:**
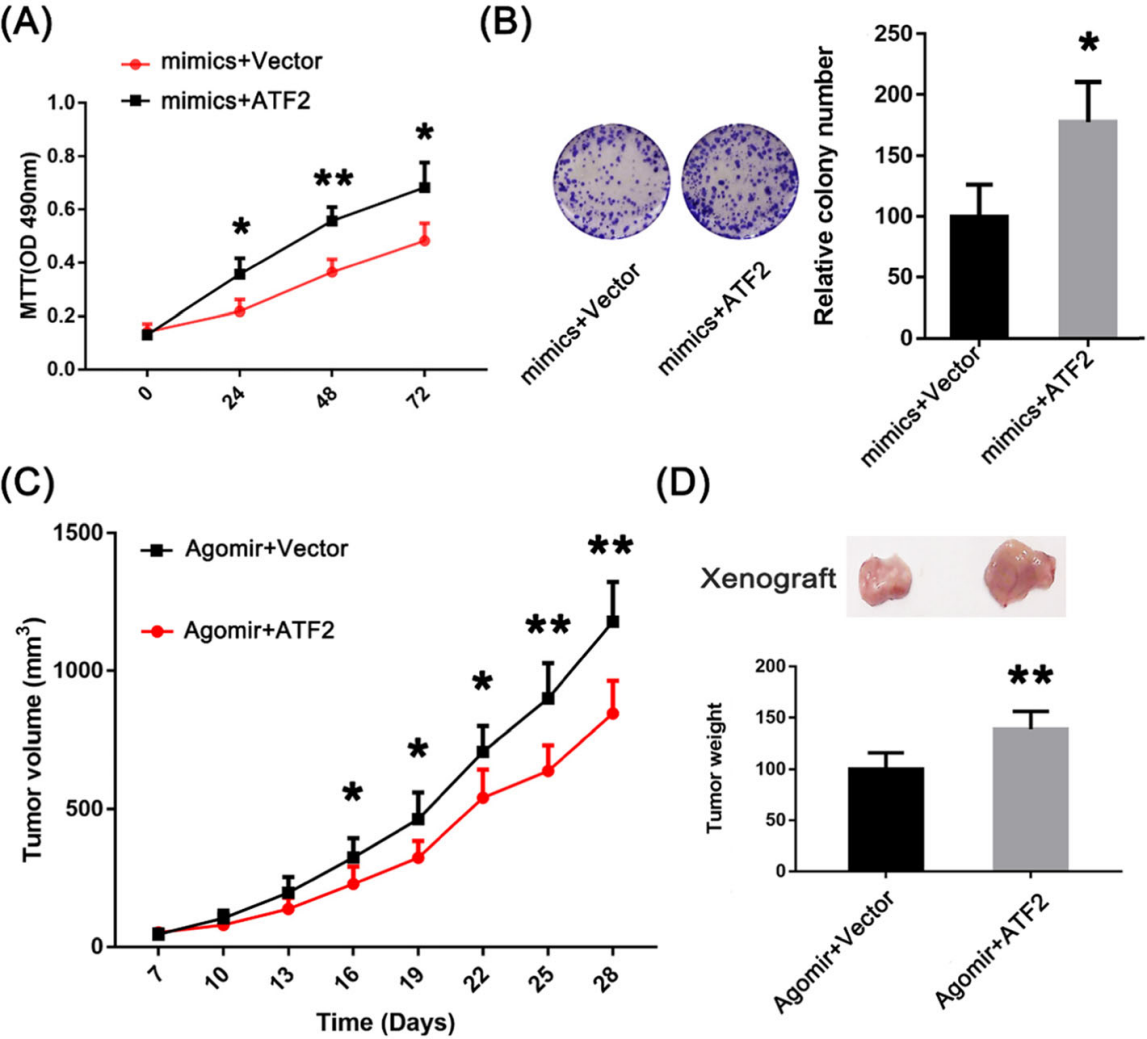
**a** After transfection of microRNA-299-5p mimics, the proliferation of ATF2-overexpressing A549 cells was significantly higher than that in the negative control group. **b** The cloning ability of ATF2-overexpressing A549 cells (mimics + ATF2) was significantly higher than that of negative control cells (mimics + vector). **c**, **d** The growth rate and weight of implanted tumours in the Agomir + ATF2 group were significantly higher than those in the AgomiR + Vector group. All results are expressed as mean ± SD. = (*p < 0.05 compared to the mimics + vector group, **p < 0.01 compared to the mimics + vector group)

We constructed A549 cells overexpressing ATF2 in order to determine whether ATF2 is involved in the proliferation of non-small cell lung cancer cells induced by microRNA-299-5p. The results showed that the proliferation and cloning ability of ATF2-overexpressing A549 cells (mimics + ATF2) was significantly higher than that of negative control cells (mimics + vector; *p* < 0.05; Fig. 10a b). The growth rate and weight of implanted tumours in the Agomir + ATF2 group were significantly higher than those in the AgomiR + Vector group (Fig. 10c d). The results showed that overexpression of ATF2 blocked the growth of non-small cell lung cancer cells by microRNA-299-5p, which demonstrated that ATF2 participates in the regulation of growth of non-small cell lung cancer cells by microRNA-299-5p.

### Overexpression of ATF2 blocks the inhibitory effect of microRNA-299-5p on the invasiveness of A549 cells

**Fig. 11:**
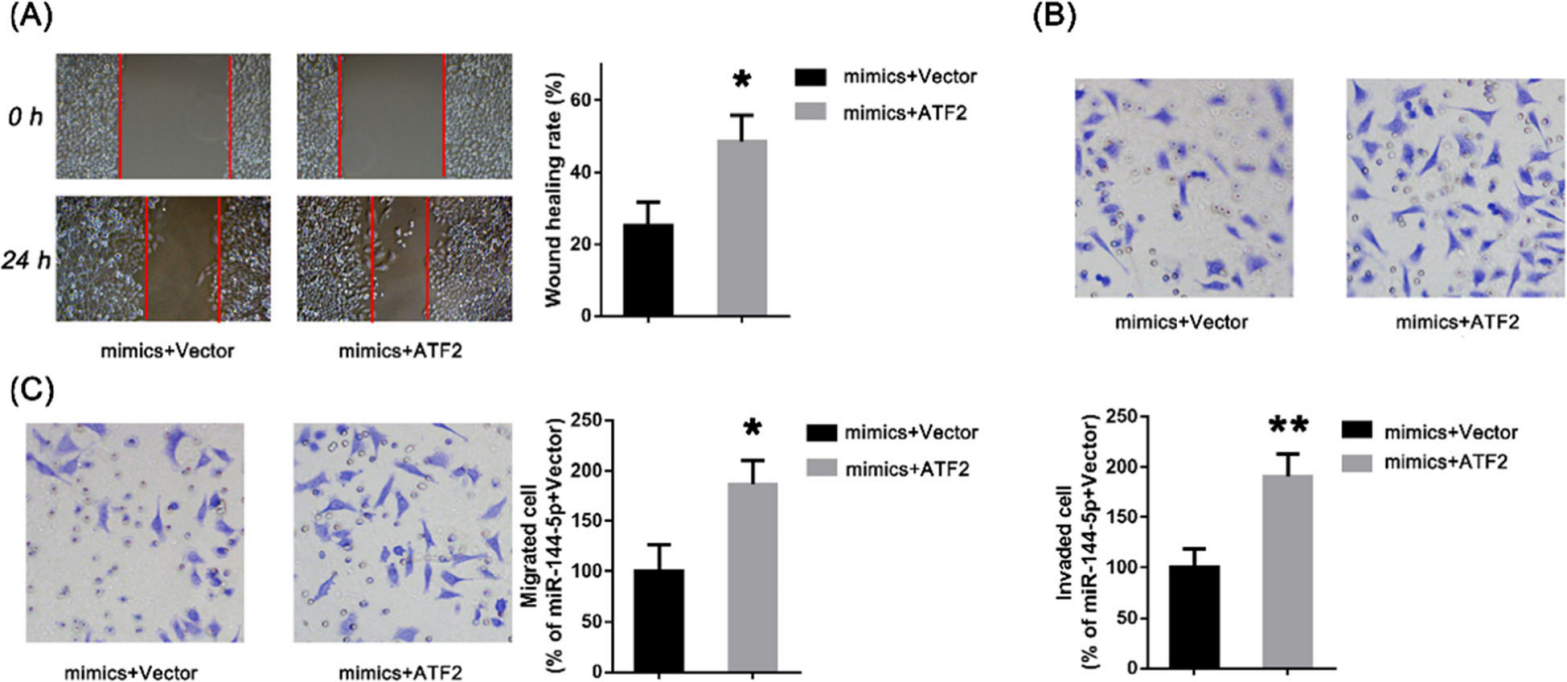
**a** The migration rate of A549 cells in the mimics + ATF2 group was significantly higher than the negative control (mimics + vector) group. **b**, **c** Transwell assays showed that that the migration and invasion ability of cells in the mimics + ATF2 group was significantly higher than in the negative control group. All results are expressed as mean ± SD (*p < 0.05 compared to the mimics + vector group, **p < 0.01 compared to the mimics + vector group)

Scratch tests showed that the migration rate of A549 cells overexpressing ATF2 (mimics + ATF2) was significantly higher than that of negative controls (mimics + vector; *p* < 0.05; Fig. 11a). Transwell assays also showed that the migration and invasion ability of cells in the mimics + ATF2 group was significantly higher than that of the negative control group (Fig. 11b c). The results showed that overexpression of ATF2 could block the effect of microRNA-299-5p on the metastasis of non-small cell lung cancer cells, which demonstrated that ATF2 may be involved in the regulation of microRNA-299-5p on the metastasis of non-small cell lung cancer cells.

## Discussion

Previous studies have shown that 6′-O-galloyl paeoniflorin (GPF) possesses significant antioxidant activity because it can protect against ultraviolet B radiation or H_2_O_2_-induced accumulation of ROS in human keratinocytes, reduce oxidative stress-induced proteins, minimise lipid and DNA damage, and inhibit mitochondrial pathway-based apoptosis [21, 22]. Matsuda et al. [23] found that the galloyl moiety is an essential part of GPF involved in scavenging free radicals. Indeed, GPF is better at scavenging 1,1-diphenyl-2-picrylhydrazine radicals than alpha-tocopherol. Our previous study showed that GPF can reduce neuroinflammation and cell damage after cerebral ischemia reperfusion by inhibiting oxidative stress responses [24]. However, whether GPF exerts anti-tumour effects has not yet been reported. In the present study, we explored the cytotoxic effects of different doses of GPF on A549 and H1299 cells in vitro and in vivo. We found that GPF had no significant cytotoxic effect on the activity of human normal epithelial cell lines at 50 or 100 μM, but it had cytotoxic effect on human non-small cell lung cancer cell lines, and inhibited the cloning ability, migration and invasion of human non-small cell lung cancer cell lines in vitro and the growth of A549-implanted tumours in nude mice. The results above also showed that the inhibitory effects of GPF against non-small cell lung cancer cells were dose dependent. These data indicated that A549 and H1299 cells were sensitive to GPF treatment.

We go ahead to explore the molecular mechanism of the anti-cancer effects of GPF in the subsequent studies. There is growing evidence that miRNAs play a key regulatory role in various types of malignant tumours, including non-small cell lung cancer [25–28]. For example, miRNA-205 targets the tumour suppressor SMAD4 to accelerate cell cycle progression in non-small cell lung cancer cells [9]. We found that miR-299-5p was altered significantly before and after GPF stimulation base on deep sequencing, hence we selected this miRNA for subsequent analysis.

In terms of cancer metabolism, AMPK acts as a tumor suppressor through several ways. MTOR is an important target of AMPK, and much effort has been expended for treatment of cancer [29]. AMPK can also regulate p53 and the activity of transcription factors and co-regulatory factors, thereby controlling the cell cycle [30, 31]. AMPK also acts as a tumour suppressor by modulating inflammation, countering metabolic changes that occur during tumorigenesis, and directly inducing cell cycle arrest [32]. In the present study, we found that GPF had cytotoxic effects on cancer cells by upregulating the expression of microRNA-299-5p via the AMPK signalling pathway.

MiR-299-5p is located in the Dlk1-Dio3 region on chromosome 14q32.31 [33]. This region is one of the largest clusters of microRNAs in the human genome, containing 54 microRNAs [33]. Studies have shown that microRNAs in the DLK1-DIO3 region are involved in the pathogenesis of many diseases, including cancer. MicroRNA-299-5p is downregulated in many malignant tumours such as metastatic breast cancer [34], neuroblastoma [35], oral squamous cell carcinoma and prostate cancer [36]. The expression and function of microRNA-299-5p in lung cancer is not yet clear. Some studies have shown that resveratrol can upregulate the expression of microRNA-299-5p in human non-small cell lung cancer cell line A549 [37]. In the present study, we found that the proliferation and cloning ability of A549 and H1299 cells transfected with an agonist of microRNA-299-5p were significantly inhibited. Experiments also confirmed that the growth rate of non-small cell lung cancer in nude mice could be significantly inhibited by microRNA-299-5p transfection. These results fully demonstrate that mirRNA-299-5p can inhibit the tumorigenesis and growth of non-small cell lung cancer cells.

ATF2 is a member of the CREB/ATF transcription factor family, and members possess a leucine zipper domain [38]. These proteins contribute to various cell behaviours, including regulating cell development, proliferation and cell death, as well as cellular responses to stress signals and DNA damage [39, 40]. Evidence suggests that ATF2 is imbalanced in individuals with cancer, whereas complete loss of ATF2 in somatic cells leads to cell death [39, 41, 42]. ATF2 is overexpressed in non-small cell lung cancer cells [43], and studies have shown that ATF2 increases the resistance of non-small cell lung cancer to cisplatin and radiotherapy by repairing DNA [44, 45].

In the present work, ATF2 was selected as a potential target of microRNA-299-5p based on gene prediction to study the regulation of ATF2 expression by microRNA-299-5p in non-small cell lung cancer cells. ATF2 was confirmed to be a direct target of microRNA-299-5p. The results of luciferase assays and western blotting confirmed that microRNA-299-5p could indeed inhibit the translation of the ATF2 3′-UTR fragment. Although ATF2 is a downstream target of microRNA-299-5p, whether it is involved in the tumorigenic effect of microRNA-299-5p needs further confirmation. Therefore, we transfected ATF2-overexpressing A549 cells with miR-299-5p. The results showed that the proliferation and metastasis abilities of ATF2-overexpressing A549 cells transfected with agomiR-299-5p were significantly higher than negative control A549 cells. These results suggest that overexpression of ATF2 can block the anti-tumor effect of microRNA-299-5p on non-small cell lung cancer cells. This indirectly proves that ATF2 is an important downstream target for microRNA-299-5p through which the biological characteristics of non-small cell lung cancer are altered.

## Conclusions

In summary, GPF has cytotoxic effects on non-small cell lung cancer cells, which also inhibits the metastasis of non-small cell lung cancer cells and upregulates the expression of miR-299-5p in A549 cells via the AMPK signalling pathway. MiR-299-5p binds to the 3′-UTR of the ATF2 mRNA and prevents gene expression, thereby inhibiting the proliferation and metastasis of non-small cell lung cancer cells. This study highlights the relationship between growth and metastasis of non-small cell lung cancer and miR-299-5p and provides a potential therapeutic target for treating non-small cell lung cancer.

## Data Availability

The datasets used and/or analyzed during the current study are available from the corresponding author on reasonable request.

## Abbreviations

AMPK: cAMP-activated protein kinase
ATF2: Activating transcription factor 2
DMEM: Dulbecco’s modified Eagle medium
DMEM: Dulbecco’s modified Eagle’s medium
DMSO: Dimethyl sulphoxide
GPF: 6′-O-galloylpaeoniflorin
MTT: 3-(4,5)-dimethylthiahiazo (−z-y1)-3,5-di-phenytetrazoliumromide
NSCLC: Non-small cell lung cancer
PBS: Phosphate-buffered saline
PIK3R2: Phosphatidylinositol 3-kinase regulatory subunit beta
qRT-PCR: Quantitative real-time PCR
rAAV: Recombinant adeno-associated virus
SD: Standard deviation
SPF: Specific pathogen-free
